# Supplementary material for: Beneficial effects of benfotiamine, a NADPH oxidase inhibitor, in isoproterenol-induced myocardial infarction in rats
Source: PLoS One. 2020 May 8;15(5):e0232413. doi: 10.1371/journal.pone.0232413 (PMC7209119; doi:10.1371/journal.pone.0232413)
Supplement: Attachment — Submitted filename: response to reviewers.doc [file pone.0232413.s001.doc]

**Rebuttal letter**

**Ref: PONE-D-19-33705**

**Title:** Beneficial effects of benfotiamine, a NADPH oxidase inhibitor, in isoproterenol-induced myocardial infarction in rats

**Journal: PLOS ONE**

We sincerely thank the Academic Editor and the respectable reviewers for their precious time and constructive criticisms. Their valuable comments were of great help in revising the manuscript and they have definitely improved it.

Accordingly, the revised manuscript has been systematically enhanced with new information and additional interpretations. Following are the detailed responses to the reviewers' and Editor's comments:

**Journal Requirements:**

1) Please ensure that your manuscript meets PLOS ONE's style requirements, including those for file naming.

**Response: All files’ names were revised**

2) At this time, we ask that you please provide the product number and any lot numbers provided with the Isoproterenol-HCl and benfotiamine purchased from Sigma-Aldrich in the study."

**Response: Done and highlighted**

3) At this time, we request that you please report additional details in your Methods section regarding animal care, as per our editorial guidelines:

(a) Please include the method of euthanasia

**Response: This part was clarified and highlighted in the manuscript**

(b) Please describe the care received by the animals, including the frequency of monitoring and the criteria used to assess animal health and well-being.

**Response: This part was clarified and highlighted in the manuscript**

4) We ask that you please provide scale bars on the microscopy images presented in Figure 5 and refer to the scale bar in the corresponding Figure legend.

**Response: This part was clarified and highlighted in the manuscript**

5) Please include captions for your Supporting Information files at the end of your manuscript, and update any in-text citations to match accordingly. Please see our Supporting Information guidelines for more information.

**No supporting information files are present in our submission**

**Review Comments to the Author**

**Reviewer one:**

1. Figures may be improved by more concise denomination of groups eg control, Iso-A, Iso-A+BT, Iso-B, Iso-B+BT instead of "prophylactic" or "treatment".

**We thank the reviewer for his/her important comment. These parts were modified in all figures**

2. For the measurement of parameters ELISA or colorimetric assays were used. Specify and sensitivity should be given for each test.

**These parts were revised and highlighted in the manuscript**

3. It is necessary to state the n (number of samples) used in each essay.

**Clarified and highlighted in the manuscript**

4. Detailed figure legends are missing and should be provided.

**Detailed figure legends were provided and highlighted at the end of manuscript after references section**

5. The reviewer would appreciate a detailed discussion of the 2010 results in comparison of the presented results. I feel these data should be considered as well. Kadare R et al. JMCC, 49, 625-538.

**This part was added and highlighted in the manuscript**

**Reviewer two:**

1) A discussion of the method of ischemia induction used here, versus Langendorff or coronary artery ligation used in other studies, should be included - specifically how the methods would impact results.

**The part was provided and highlighted in the manuscript**

2) As NOX is a central molecule in the study, the measurement of NOX enzyme content should be supplemented by activity assays. Determination of the NOX subunits and **isoforms may also add value.**

**NADPH oxidase activity is a relatively nonspecific activity displayed by several electron transfer enzymes and dehydrogenases as well. Therefore, assessing the NADPH oxidase activity of whole cell homogenates will not be accurate due to being heavily contaminated by all mitochondrial and cytosolicdehydrogenases**(Laurindo, Fernandes & Santos 2008)**. Regarding NOX subunits, this would merit further investigation in a future study.**

3) More detailed descriptions of the methods used is needed.

**Response: More detailed methodology for ECG and histopathology were provided and highlighted in the manuscript. ELISA kits used in the present study employed Double Antibody Sandwich Technique. This part was added and highlighted in the manuscript. On the other hand, detailed procedures provided by ELISA and colorimetric kits are usually not written as each differs from one company to another. Thus, it is usually written that procedures were done according to the manufacturer's instructions.**

4) Why was PKC used as a marker of inflammation but not one of the "conventional" markers (e.g. IL-6 or TNF alpha)? Also, which PKC isoforms play a role?

**PKC activation mediates inflammatory response signals through NF-κB pathway** (Kim et al. 2013)**. The activation of NF-κB is responsible for the upregulation of inflammatory cytokines e.g. IL-6 or TNF alpha** (Kany, Tilmann Vollrath & Relja 2019)**. Therefore, its elevation is an indicator of increase in the levels of inflammatory cytokines. Furthermore, ROS, produced through NADPH oxidase, activates NF-kB through various intermediates, including PKC** (Panday et al. 2015)**. In the present study, total PKC was estimated which was previously shown to participate in cardiac remodeling, oxidative stress and arrhythmogenesis.**

5) Were all the data normally distributed?

**Response: Provided and added in the manuscript.**

6) Please indicate p-values when reporting significant differences in text.

**Response: Added and highlighted in the manuscript.**

7) A measurement of NADPH levels in the cardiac tissue may add an explanation for the results observed. This may facilitate a mechanistic explanation for the link between BFT and decreased NOX activity.

NADPH is an important cofactor for NADPH oxidase. NADH and NADPH are very labile which would make their extraction very difficult for measurements by fluorescence and enzymatic methods(You et al. 2019).

8) The manuscript may benefit from language editing as some grammatical errors occur throughout the text.

Concerns the language editing, the manuscript has been subjected to peer-revision by Nature Research Editing Service and the certificate is attached with the submitted files.

**References:**

Kany, S., Tilmann Vollrath, J. & Relja, B. 2019, ‘Molecular Sciences Review Cytokines in Inflammatory Disease’, *International Journal of Molecular Sciences*, vol. 20, no. 6008, viewed 28 January 2020, <www.mdpi.com/journal/ijms>.

Kim, H., Zamel, R., Bai, X.H. & Liu, M. 2013, ‘PKC Activation Induces Inflammatory Response and Cell Death in Human Bronchial Epithelial Cells’, *PLoS ONE*, vol. 8, no. 5.

Laurindo, F.R.M., Fernandes, D.C. & Santos, C.X.C. 2008, ‘Chapter 13 Assessment of Superoxide Production and NADPH Oxidase Activity by HPLC Analysis of Dihydroethidium Oxidation Products’, *Methods in Enzymology*, vol. 441, Academic Press Inc., pp. 237–60.

Panday, A., Sahoo, M.K., Osorio, D. & Batra, S. 2015, ‘REVIEW NADPH oxidases : an overview from structure to innate immunity-associated pathologies’, *Cellular & Molecular Immunology*, vol. 12, no. August 2014, pp. 5–23.

You, S.-H., Lim, H.-D., Cheong, D.-E., Kim, E.-S. & Kim, G.-J. 2019, ‘Rapid and sensitive detection of NADPH via mBFP-mediated enhancement of its fluorescence’, *PLOS ONE*, vol. 14, no. 2, viewed 28 January 2020, <https://doi.org/10.1371/journal.pone.0212061.g001>.
